# Differential expression of cytokines and receptor expression during anoxic growth

**DOI:** 10.1186/s13104-018-3520-5

**Published:** 2018-06-25

**Authors:** Balbina J. Plotkin, Ira M. Sigar, Julie A. Swartzendruber, Amber Kaminski, James Davis

**Affiliations:** Department of Microbiology and Immunology, Midwestern University, Downers Grove, IL 60515 USA

**Keywords:** Anoxia, Anaerobic, Cytokine, IL-6, IL-8, IL-11, Cell receptors, Metastasis, Secretomic profile

## Abstract

**Objective:**

Cell density in tumor cell three dimensional (3D) cultures affects secretome expression of components. A microenvironment characteristic shared by high-density 3D cell culture and in vivo tumor masses is poor oxygenation, with anoxia being a natural cell state in tumor centers. Until recently, the ability to study anoxia-adapted cell physiology was not possible. Using a newly-developed methodology, anoxic HeLa cell secretome expression was measured.

**Results:**

Anoxic HeLa cell cytokine levels after 3 days’ (hypoxia inducible factor, *HIF1* positive) and 10 days’ growth (*HIF1* negative; anaerobic respiration) were significantly (*p* < 0.01) higher than normoxic controls for: IL-8 (1.8- and 3.4-fold higher, respectively), GRO (1.3- and 1.1-fold higher, respectively), and IL-11 (1.4- and 1.1-fold higher, respectively). In contrast, G-CSF, IFNα2, and CXCL-10 levels decreased over time (day 3 vs. day 10). Thus, metabolically active HeLa cells respond to the lack of oxygen, in part, by regulating the levels of cytokines produced. Cytokines expressed at increased levels, in the absence of oxygen, correspond to a secretomic profile reported for paracrine signaling pathways associated with metastasis. Further studies defining physiologic changes that occur upon anoxic growth may lead to the discovery of novel chemotherapeutic drug targets.

**Electronic supplementary material:**

The online version of this article (10.1186/s13104-018-3520-5) contains supplementary material, which is available to authorized users.

## Introduction

Intercellular communication is an essential element in modulating physiological processes in normal and abnormal tissue. The chemical signaling compounds responsible for regulation of various metabolic phenotypes triggers changes in cell proliferation and density, as well as cell migration. These adaptations can occur in response to the synergistic signaling pathway activations by interleukin 6 (IL-6) and interleukin 8 (IL-8). The IL-6/IL-8 mediated pathway activations result in cell phenotypic alteration with display of dendritic protrusions by monolayer cells and accompanying increased cell migration. In our initial study detailing cell characteristics upon long-term growth (17 days) in the absence of oxygen, we observed cell morphologic changes similar to that described for human fibrosarcoma (HT1080) cells and triple negative metastatic breast cancer (MDA-MB-231) cells in high cell density three dimensional (3D) cultures [1–3]. In addition, these studies indicate that the secretome profiles of HT1080 cells and MDA-MB-231 are altered during the metastatic process [1, 2, 4–6]. The metastatic-relevant cytokines produced under these environmental conditions were IL-6 and IL-8 [2]. To date, evidence indicates that both cytokines are necessary and sufficient for cell morphology changes (dendritic projection appearance) and enhanced cell migration, a process important in tumor cell metastasis, i.e., 3D vs. two dimensional (2D) culture conditions. However, a factor not taken into consideration that is associated with increased cell density is a reduction in available oxygen.

Oxygen depletion is analogous to the low to absent oxygen levels present in solid tumors, which occurs both as a result of high cell density and reduced vascularization. The direct contribution of oxygen on cytokine production, particularly IL-6/IL-8 is not known. Using a method developed in this lab for the long-term cultivation of mammalian cells in the absence of oxygen, the expression of IL-6 and IL-8, as well as additional pro- and anti-inflammatory cytokines by anaerobically-adapted HeLa 229 cells was determined using multiplex analysis.

## Main text

The ability of anaerobically-cultured cells to express their secretome is not known. To determine whether HeLa 229 cells can engage in anaerobic synthetic metabolism to produce members of its secretome, and if that production differs from normoxic culture expression, culture supernatants (n = 19) from cells were tested after 3 and 10 days’ incubation in the absence of oxygen. To confirm metabolic status, hypoxia inducible factors 1α (*HIF1*α) mRNA and protein expression at day 3 anoxic cell incubation (glycolytic fermentation, *HIF1α* positive), and day 10 anoxic cell incubation (anaerobic respiration, *HIF1α* negative) were performed and confirmed, as previously described [3]. As previously reported, we found no differences in *HIF1α* expression measured, i.e., at day 3, cells were *HIF1α* positive and at day 10, cells were negative for HIF expression [3]. ROS production at day 10 anoxic cultivation was also confirmed using the cell permeant reagent 2′,7′-dichlorofluorescein diacetate (DCFDA) according to manufacturer’s specifications (Abcam), as previously described [3]. Chemokine levels were measured using Milliplex^®^ MAP Human Cytokine/Chemokine Magnetic Bead Panel and Bio-Plex MAGPIX Multiplex Reader according to manufacturer’s protocol. HeLa 229 cells were prepared and cultured in the absence of oxygen, as previously described [3]. Briefly, HeLa 229 cells that were seeded normoxically (5% CO_2_ in air) for 24 h in growth media (DMEM: 4.5 g/L glucose, 10% FBS, 50 µg/mL gentamicin; 1.68 × 10^5^ cells/well of a 24 well plate) were then placed in an anaerobic chamber (Whitley A35), and medium was immediately replaced with de-gassed PS-74656 medium. Medium of normoxic controls was also replaced with atmospheric PS-74656 medium and incubation continued in 5% CO_2_ in air. After incubation, cell supernatants (n = 19) were removed and immediately frozen (− 80 ^°^C) until testing. Statistical analysis was performed by two-way ANOVA (GraphPad Prism). Where appropriate, Tukey post hoc tests were performed.

**Fig. 1:**
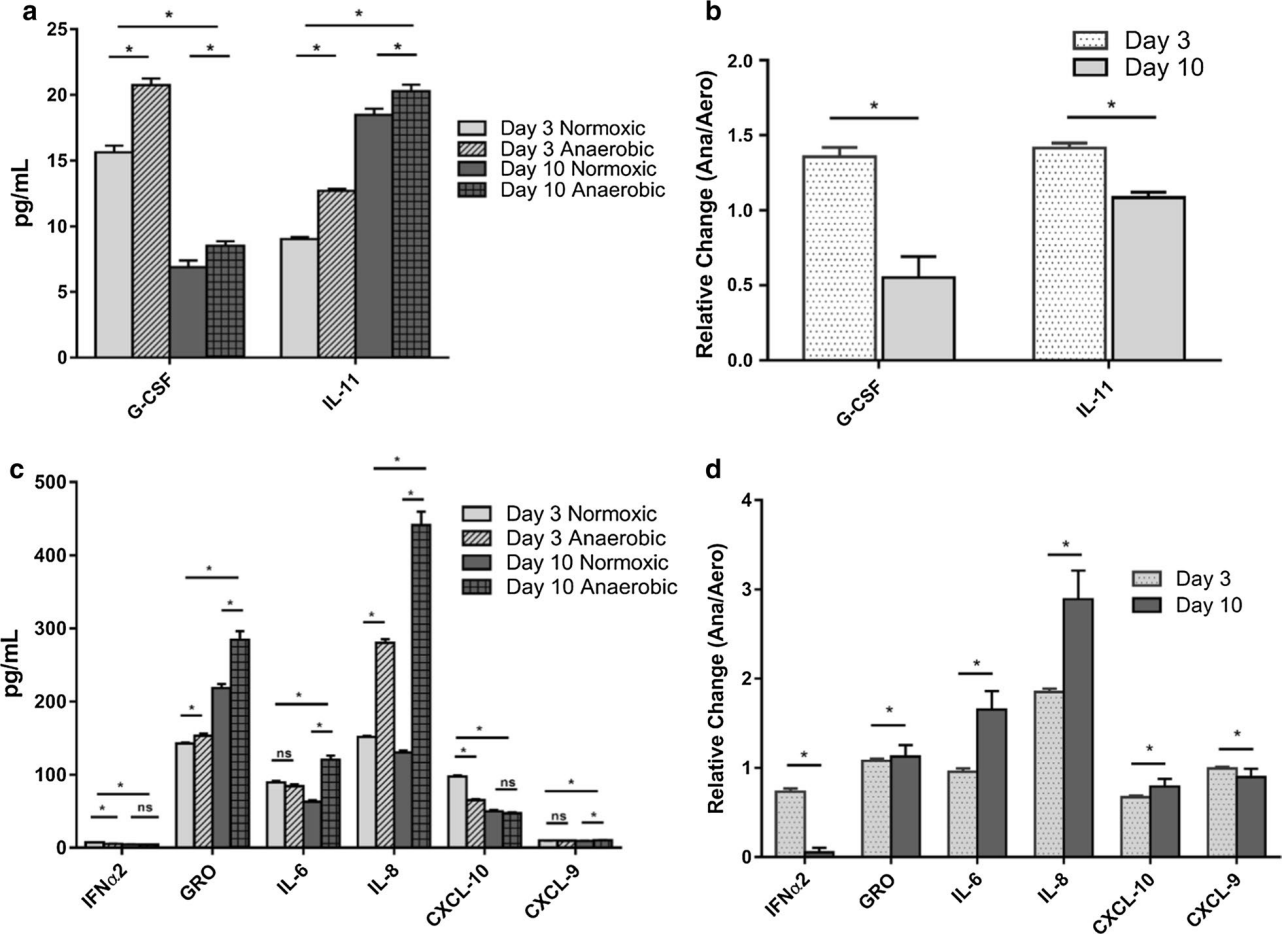
Cytokine production after 3 days (*HIF1* positive) or 10 days (*HIF1* negative) under anaerobic growth conditions as compared to normoxic (5% CO_2_) controls. **a** Levels of anti-inflammatory cytokine production; **b** ratio of anti-inflammatory cytokine production under anaerobic conditions to normoxic controls (relative change); **c** levels of pro-inflammatory cytokine production; **d** ratio of pro-inflammatory cytokine production under anaerobic conditions to normoxic controls (relative change). Results are shown as the mean ± standard error of the mean (n = 19; *p < 0.01) [7]

HeLa 229 cells differentially express both pro- and anti-inflammatory cytokines dependent on levels of oxygen present (anoxic vs. normoxic) and amount of time cells were anoxia adapted (Fig. 1, Table 1). Anoxic HeLa cell cytokine levels after 3 days’ (*HIF1* positive) and 10 days’ growth (*HIF1* negative, ROS positive) were significantly (*p* < 0.01) higher than normoxic controls for: IL-8 (1.9- and 3.4-fold higher, respectively), GRO (1.1- and 1.3-fold higher, respectively), and IL-11 (1.4- and 1.1-fold higher, respectively). In contrast, granulocyte colony stimulating factor (G-CSF), interferon alpha 2 (IFNα2), and CXCL-10 levels decreased over time (day 3 vs. day 10). No production of Eotaxin, interferon gamma (IFN-γ), IL-4, IL-10, IL-12(p40), IL-12(p70) or IL-13 was detected. The significantly (*p *< 0.01) increased expression of IL-6 and IL-8 upon anoxic culture and over time in the absence of *HIF1* is particularly relevant since IL-6/IL-8 expression is linked to enhanced cell growth and survival, as well as neutrophil chemotaxis and risk for metastasis in prostate, lung, liver, breast and colon cancers [2, 4, 7–9].

**Table 1:** Effect of anoxic culture on expression of pro- and anti-inflammatory cytokines in the presence (day 3) and absence (day 10) of *HIF1* expression

Cytokines	Relative change (anoxic/normoxic)^a^ Day 3^b^	Relative change (anoxic/normoxic)^a^ Day 10^b^
IFN-α2	0.7^c d^	0.9
GRO	1.1^c d^	1.3^c^
IL-6	0.9^d^	1.9^c^
IL-8	1.9^c d^	3.4^c^
CXCL-10	0.7^c d^	0.9
CXCL-9	1.0^d^	1.1^c^
G-CSF	1.3^c d^	1.2^c^
IL-11	1.4^c d^	1.1^c^

^a^Ratio anoxic cytokine levels to normoxic levels

^b^Number of days in the absence of oxygen

^c^Significant change in production (p ≤ 0.01). n = 19 replicates

^d^Significant change (p ≤ 0.01) between day 3 and day 10 relative cytokine production

**Fig. 2:**
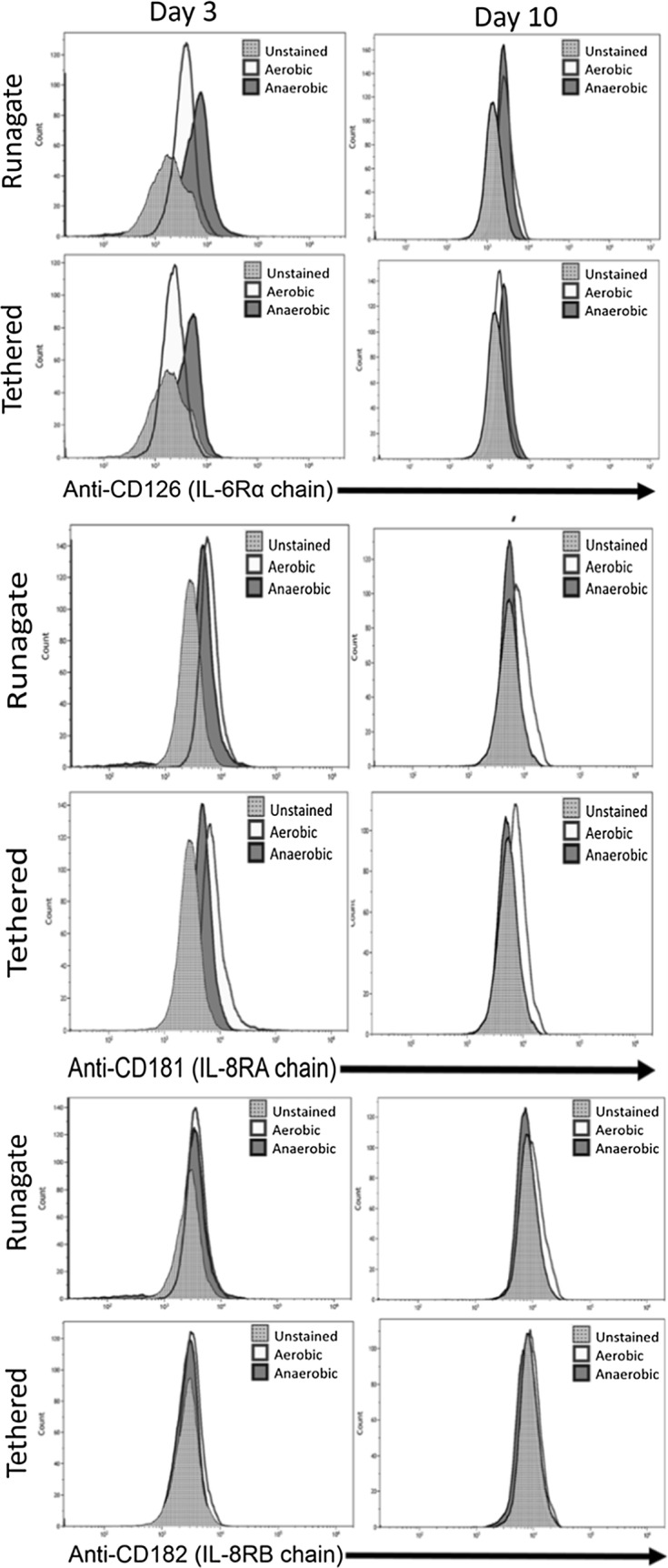
Expression of IL-6/IL-8 receptors on tethered and runagate HeLa cells upon anoxic culture. Runagate or tethered cells were separated by centrifugation and assessed for expression of IL-6 receptor (CD126/IL-6R) and IL-8 receptors (CXCR1/CD181/IL-8RA and CXCR2/CD182/IL-8RB). Runagate or tethered cells were assessed at day 3 and day 10 of culture under aerobic or anaerobic growth conditions using three-color flow-cytometry. Histograms show the receptor expression in unstained cells (fluorescence minus one) or cells cultured aerobically or anaerobically

IL-6/IL-8 can have autocrine and paracrine activity [6, 10]. Variable receptor expression is critical in dynamic cell communication and interaction with their environment. Detection of altered IL-6/IL-8 receptors expression upon anoxic growth would indicate not only that cells can differentially express cell receptors under anaerobic conditions, but that autocrine activity of IL-6/IL-8 has important implications for the function of cells in the absence of oxygen. We measured the expression of IL-6/IL-8 receptors by flow cytometry to determine if the capability of HeLa cells to respond to autocrine signaling under anaerobic conditions is altered (Fig. 2). Detection of the presence of IL-6 and IL-8 receptors on day 3 and day 10 HeLa cells cultured under aerobic (monolayer and suspension cells) and anaerobic (tethered and runagate cells) conditions was performed by flow cytometry. Tethered and runagate cells represent the phenotypically distinct morphologies of anaerobically cultured cells. Tethered cells are attached cells with dendritic projections and a smaller cell size; runagate cells are rounded cells in suspension with a smaller cell size and decreased cytoplasm to nucleus ratio [3]. Runagate or tethered cells were separated by centrifugation and fixed for 20 min in 3.7% paraformaldehyde. IL-6 signals through a single IL-6 receptor (CD126/IL-6R), while IL-8 signals through two receptors (CXCR1/CD181/IL-8RA and CXCR2/CD182/IL-8RB). Therefore, to screen for IL-6/IL-8 receptors, cells were incubated with anti-CD16/CD32 (Biolegend) for 20 min at 4 °C in FACS buffer (1XPBS, 5%FBS). This was followed by incubation for 30 min at 4 ^°^C with anti-CD162 (APC, Biolegend), anti-CD181 (FITC, Biolegend), and anti-CD182 (PE, Biolegend). Cells were washed in FACS buffer and centrifuged to remove unbound antibody and analyzed on the Beckman Coulter CytoFLEX. Staining controls included the use of OneComp eBeads (eBioscience) to determine positive signal for each antibody (anti-CD162, anti-CD181 and anti-CD182), along with unstained OneComp eBeads and unstained HeLa cells to determine negative signal (Additional file 1: Figure S1).

The expression of anoxically-produced IL-6 and IL-8 correlated to increased expression of specific receptors (Fig. 2). This indicates that anaerobically-adapted HeLa cells respond to the autocrine signaling of both IL-6 and IL-8. After 3 days of culture under anaerobic conditions, we found elevated expression of CD126, the alpha chain of the IL-6 receptor, in both runagate and tethered cells. CD181 and CD182, IL-8 receptor A and B expression levels were not elevated on day 3 when compared to expression under aerobic conditions, and CD181 expression was lower in anaerobic tethered cells. After 10 days of culture, CD126 and CD182 expression was similar between aerobic and anaerobic cells, while CD181 had lower expression under anaerobic conditions as compared to aerobic conditions.

This work shows that anoxia-adapted HeLa cells are metabolically active and they respond to the lack of oxygen by adjusting the levels of cytokines produced. Cytokines expressed at increased levels correspond to secretomic profiles reported for paracrine signaling pathways associated with metastasis. Additionally, we found evidence that the altered secretomic profile under anoxia may be influenced by the autocrine signaling of IL-6 through the IL-6 receptor. Autocrine signaling of IL-6 is known to enhance survival and proliferation of tumors [1, 5, 11]. Further studies defining physiologic changes that occur upon anoxic growth may lead to the discovery of novel chemotherapeutic drug targets.

## Limitations

Analysis was focused on secretome expression for those cytokines previously reported to be expressed by HeLa cells, as we indicated in the main text. There was additional focus with respect to their potential autocrine activity, on quantifying cytokine receptors described to play a role in metastasis and tumor formation, i.e. IL-6/IL-8. We achieved our objective of determining whether anoxic-adapted HeLa cells are capable of synthesizing and excreting chemokines as well as exhibiting differential receptors expression. Although anaerobic attached cells (tethered cells) exhibited the dendritic characteristic of tumor cell migration, actual migration was not directly measured. Thus, the tumorigenic capability of aerobic vs. anoxic-adapted cells can only be hypothesized at this point. Another limitation is that while anaerobically cultured cells differentiate into attached cells (tethered cells) and cells in suspension (runagate cells), measurement of cytokine levels was for the culture *in toto* (both cell phenotypes). We prefered to test the entire cell anoxic cell population in order to maximize our sample size.

## Additional file

**Additional file 1: Figure S1.** Additional figure positive control; flow cytometry positive control; positive controls for the flow cytometry cytokine receptor experiments.

## Abbreviations

IL: interleukin
3D: three dimensional
2D: two dimensional
HIF: hypoxia inducible factor
GRO: CXCL1 chemokine
DCFDA: 2′,7′-dichlorofluorescein diacetate
G-CSF: granulocyte colony stimulating factor
IFNα: interferon alpha
IFNγ: interferon gamma
