# Supplementary material for: Differential expression of cytokines and receptor expression during anoxic growth
Source: BMC Res Notes. 2018 Jun 25;11:406. doi: 10.1186/s13104-018-3520-5 (PMC6019713; doi:10.1186/s13104-018-3520-5)
Supplement: Supplementary file 1 — Additional file 1: Figure S1. Additional figure positive control; flow cytometry positive control; positive controls for the flow cytometry cytokine receptor experiments. [file 13104_2018_3520_MOESM1_ESM.pdf]

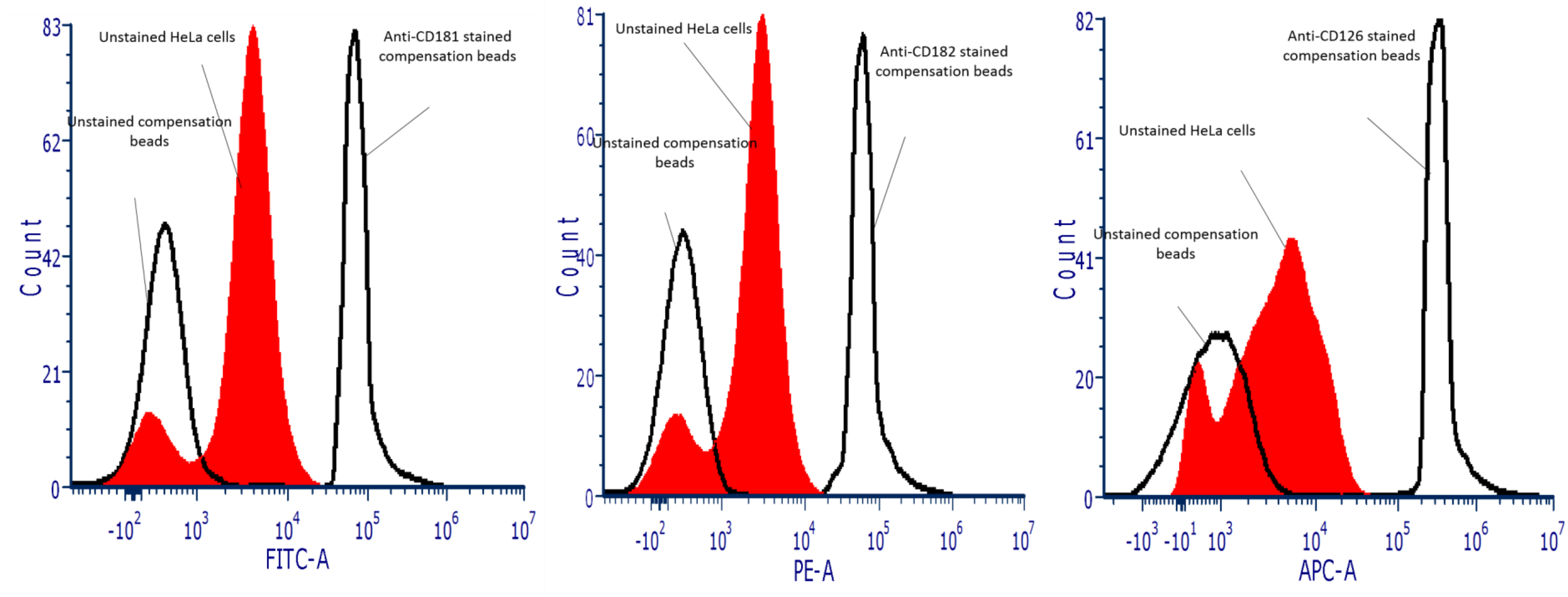

Figure S1. Staining controls for expression of IL-6 and IL-8 receptors. OneComp eBeads (compensation beads from eBioscience) were used as a positive control to demonstrate successful binding of fluorochrome-conjugated antibodies in comparison to HeLa cells and OneComp eBeads incubated without fluorochrome-conjugated antibody. Left panel shows positive staining for FITC-conjugated anti-CD181 (IL-8RA chain). Middle panel shows positive staining for PE-conjugated anti-CD182 (IL-8RB chain). Right panel shows positive staining for APC-conjugated anti-CD126 (IL-6Rα chain).
